# Identification and quantification of phenolic and fatty acid profiles in *Pinus halepensis* mill. seeds by LC‐ESI‐MS and GC: Effect of drying methods on chemical composition

**DOI:** 10.1002/fsn3.2151

**Published:** 2021-02-25

**Authors:** Amani Mahdhi, Hanene Ghazghazi, Meriem El Aloui, Ridha Ben Salem, Ghayth Rigane

**Affiliations:** ^1^ Laboratory of Management and Valorization of Forest Resources LR161INRGREF01 Ariana Tunisia; ^2^ National Institute for Research in Rural Engineering Water and Forest (INRGREF) University of Carthage Tunis Tunisia; ^3^ Laboratory of Organic Chemistry LR17ES08 Sciences Faculty of Sfax University of Sfax Sfax Tunisia; ^4^ Chemistry‐Physics Department, Sciences and Technology Faculty of Sidi Bouzid University of Kairouan Sidi Bouzid Tunisia

**Keywords:** bioactive secondary metabolites, biological activities, fatty acids, *Pinus halepensis*

## Abstract

This study aimed to evaluate *Pinus halepensis* Mill. seeds oil as well as methanolic‐aqueous extract on fatty acid and phenolic's composition as well as antioxidant activities with regard to the cones drying methods: convection and sun methods. The highest amounts of total phenols (14.63 ± 0.05 mg GAE/g DW), flavonoids (3.3 ± 0.02 mg QE/g DW), and condensed tannins (0.36 ± 0.05 mg CE/g DW) were showed in the seeds obtained by sun‐dried method. Methanolic‐aqueous seeds extracts were subjected to LC‐ESI‐MS analysis in order to identify and quantify the phenolic composition. This technique allowed us to identify eleven phenolic compounds: two phenolic acids and nine flavonoid compounds such as cirsiliol, catechin (+), luteolin, and luteolin‐7‐*O*‐glucoside, which were present in the two studied samples while apigenin, naringenin, and cirsilineol were only identified in the extract obtained from sun‐drying method seeds. The chemical components of the oils were analyzed using GC, and significant differences were found between the two studied seeds oil (*p* < .05). Furthermore, the antioxidant activities were investigated using DPPH and ABTS.^+^ assays. The results showed that the methanolic‐aqueous extract from seeds dried by sun method had the highest antioxidant activities (0.08 and 0.05 mg/ml, respectively). This study could provide useful information for industry to produce potentially bioactive plant extract.

## Graphical abstract

Minirel contents of *Pinus halepensis* Mill. seeds were present in significant amounts and very rich in K, Na, Ca and Fe. On the other hand, cirsiliol, catechine (+), luteolin and luteolin‐7‐O‐glucoside were present in the two studied samples while apeginin, naringenin and cirsilineol were only identified in the extract obtained from sun drying method seeds’. These results may be attractive for various commercial purposes as food supplements and herbal medicine products.

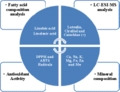

## 1 INTRODUCTION

The genus *Pinus halepensis* belonging to the *pinacea* family is one of about 800 *Pinus species*. *Pinus halepensis*is original in the Northern Hemisphere including the Mediterranean region, Caribbean area, Asia, Europe, as well as North and Central American (Bello‐Rodríguez et al., 2020; Rigane et al., 2019). Pines are widely used in the traditional therapeutic and pharmaceutical treatment practiced in the world. Several pine *species* also know other uses in the aromatic and cosmetic field under to the essential oils contained in the needles (Djerrad et al., 2015; Hamrouni et al., 2014; Kadri et al., 2015; Rigane et al., 2019).

For many centuries, many Arabic countries used *P. halepensis* seeds for preparing a sweet pudding which was called “Assida‐Zgougou.” Recently, it has been added as an ingredient in ice creams and candies. Therefore, for food industry, it will be very important to know the best cones dried methods in point of view chemical composition of the obtained seeds including fatty acids and phenolic compounds which are important components for its contribution as nutritional values.

To the authors’ knowledge, no comparative work has been published on chemical composition and antioxidant activity of *P. halepensis* oil as well as methanolic‐aqueous extract with respect to the dried cones methods which include convection and sun methods. Therefore, the aim of this work was to characterize *P. halepensis* seeds oil and methanolic‐aqueous extract using the following parameters: fatty acid profiles, mineral composition, identification and quantification of individual phenolic compounds present in the methanolic‐aqueous seeds extract using LC‐ESI‐MS, colorimetric quantification of total phenol, flavonoid, and condensed tannin contents as well as the evaluation of its antioxidant activities via DPPH and ABTS assays. These data will offer a strong framework for new discoveries, particularly to the agri‐food processing industries.

## 2 MATERIALS AND METHODS

### 2.1 Plant material and Sample preparation

**FIGURE 1:**
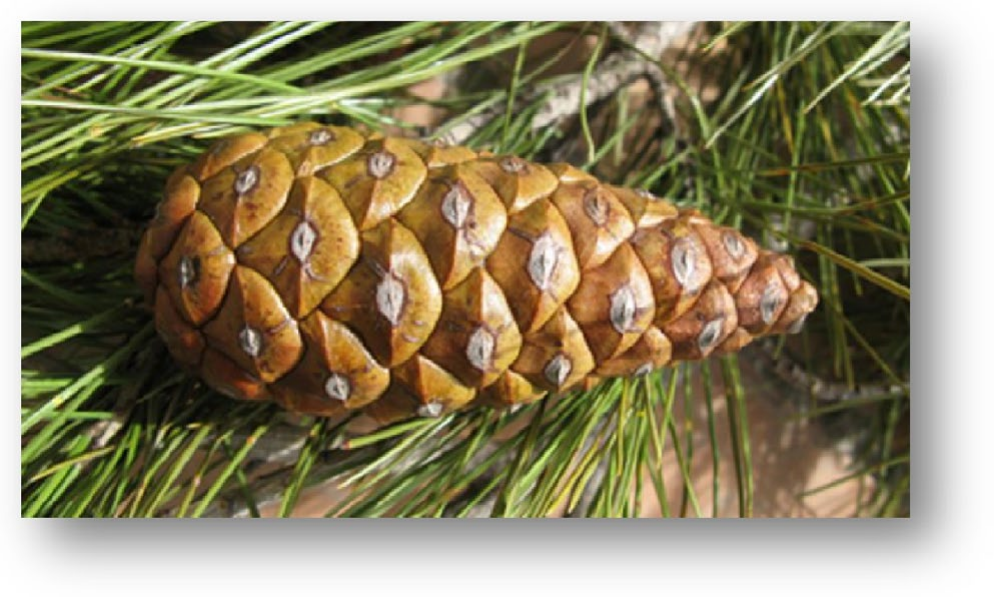
*Pinus halepensis* Mill. cones

Cones of *Pinus halepensis* (Figure 1) were collected in October 2017, from Henchir Naam arboreta from northeastern provinces of Tunisia (43°49′ N 3°24′ E, 450 m) under semi‐arid bioclimate which established in 1959. The arboretum covers a total area of 57 ha. The average annual precipitation is 509 mm/year. The mean annual temperature of the hottest months is 33.6 and 3.1°C of the coldest one. A voucher specimen of *P. halepensis* (LGVR 120) was deposited at the Laboratory of Management and Valorization of Forest Resources, Tunisia. The harvesting of pine cones was done with pruning shears on various mature trees selected at random at different heights and the four cardinal points. Transport of samples was performed in ventilated plastic boxes. The cones were dried using two methods:

- dried in an oven at 60°C for 48 hr in a domestic oven
- dried using sun energy until pine cones open

After that, seeds were finely ground using an electric grinder to get a fine powder that was kept in closed containers (vials) until the day of their analysis.

### 2.2 Extraction of plant material

#### 2.2.1 Extraction of phenolic compounds

Two grams of each *P. halepensis* seeds powder was extracted by maceration using methanol aqueous (80:20, v/v) (50 ml) for 3 hr at 80°C and under continuous agitation. The resulting extract was then filtered through Whatman no. 4 paper and centrifuged for 10 min at 4,500 *g*. The supernatant was lyophilized and stored at −21°C, until use.

#### 2.2.2 Extraction of condensed tannin (CT)

Briefly, 2 g of dry weights of each *P. halepensis* seeds powder was extracted using a Soxhlet apparatus with *n*‐hexane for 6 hr to remove apolar substances. After that, ascorbic acid solution (0.1%, w/v) has been added to the solid residue which then used in order to isolate CT using a CH_3_COCH_3_/H_2_O mixture (70:30, v/v). Subsequently, acetone solvent was evaporated using a rotary evaporator and the aqueous phase was washed successively with chloroform and ethyl acetate in order to remove chlorophyll, carotenoids, low molecular weight phenolics, and tannin monomers. The extract was filtered through Whatman no. 4 paper and then stored at −21°C until further use (Yahyaoui et al., 2019).

### 2.3 Determination of minerals

The mineral composition (Ca, Na, K, Mg, Fe, Zn, and Mn) of each dried methods *P. halepensis* seeds was carried as described before by Kadri et al. (2015).

### 2.4 Total phenols, flavonoids, and tannins contents

Total phenol content was evaluated according to F–C method according to Khedher et al., (2020) with slight modification using a UV‐Visible spectrometer (BECKMAN DU 800). Briefly, 50 µl aliquot of extract was assayed with 250 µl of phenol reagent and 500 µl of aqueous sodium carbonate (20%, w/v). The mixture was vortexed and diluted with water to a final volume of 5 ml. After incubation for 30 min at room temperature, the absorbance was measured at 765 nm. Total phenol content was calculated based on a gallic acid calibration curve (*r* ^2^ = .914) and expressed as mg gallic acid equivalents/g dry weight (DW). In addition, total flavonoid content was estimated as reported previously by Ben Hmed et al. (2020): One milliliter aliquot of the appropriately diluted sample or standard solution of quercetin was added to 10‐ml volumetric flask containing 4 ml of water. At zero time, 300 µl of NaNO_2_ (5%, w/v) in water was added to the flask. After 5 min, 300 µl of 10% AlCl_3_ was added. At 6 min, 2 ml of aqueous NaOH (1 mol/L) was added to the mixture. Immediately, the mixture was diluted with water to 10 ml. The absorbance of the mixture, characterized by a pink color, was determined at 510 nm compared to a water control. The total flavonoid content was quantified using quercetin standard curve (*r* ^2^ = .996) and expressed as mg QE/g DW. On the other hand, the CT was evaluated by the method used by Yahyaoui et al. (2019): To 50 µl of each extract 3 ml of 4% vanillin reagent and a 1.5 ml volume of 4% concentrated H_2_SO_4_ were added. After 15 min, the absorbance was measured at 500 nm. Catechin was used as a standard. The content of CT in the MeOH‐H_2_O extracts was expressed as mg CE/g DW.

### 2.5 Oil extraction

Briefly, 100 g dry weights of each *P. halepensis* seeds powder was extracted using a Soxhlet apparatus with *n*‐hexane for 10 hr at 65°C. Finally, the obtained seed oils were stored in black glass bottles and kept in a cold room at + 4°C until further use.

### 2.6 Fatty acid composition analysis

The fatty acid composition of the oils was determined using EEC 2568/1991 (1991) methods to prepare fatty acid methyl esters (FAMEs), followed by a chromatographic analysis step which was performed in a Hewlett Packard 6890 gas chromatography using a capillary column (Stabilwax, Restek, length 50 m, internal diameter 0.32 mm, and film thickness 0.25 µm). The column temperature was isothermal at 180°C,and the injector 230°C and detector temperatures were 250°C. Fatty acids were identified by comparing retention times with standard compounds including lauric (C12:0), tridecylic (C13:0), myristic (C14:0), palmitic (C16:0), hypogeic (C16:1n‐9) + palmitoleic (C16:1n‐7), stearic (C18:0), oleic (C18:1n‐9) + Z‐vaccenic (C18:1n‐7), linoleic (C18:2), linolenic (C18:3), arachidic (C20:0), gadoleic (C20:1), behenic (C22:0), and erucic (C22:1) acids expressed as percentages of fatty acid methyl esters. Results were expressed as relative percent of total area.

### 2.7 LC‐ESI‐MS analysis

The LC‐ESI‐MS/MS analysis was carried out using a LCMS‐2020 quadrupole mass spectrometer (Shimadzu, Kyoto, Japan) equipped with an electrospray ionization (ESI). The mass spectrometer was operated in negative ion mode with a nebulizing gas flow of 1.5 L/min, a dry gas flow rate of 12 L/min, a block source temperature of 400°C, a DL (dissolving line) temperature of 250°C, the full scan spectra from 50 to 2,000 m/z, and the negative ionization mode source voltage −4,500 V.

The mass spectrometer was coupled in‐line with an ultra‐fast liquid chromatography system consisting of a LC‐20 CE XR binary pump system, SIL‐20AC XR autosampler, CTO‐20AC column oven, and DGU‐20A 3 R degasser (Shimadzu). An Aquasil C18 column (Thermo Electron, Dreieich, Germany) (150 mm × 3 mm, 3 μm) preceded by an Aquasil C18 guard column (10 mm × 3 mm, 3 μm, Thermo Electron) was used for the analysis. The solvents used were as follows: **A** (0.1% formic acid in H_2_O, v/v) and **B** (0.1% formic acid in methanol, v/v). The elution gradient established was as follows: 0 min—10% **B**, 45 min—100% **B**, from 45 to 55 min, 100% **B**. Re‐equilibration duration was 5 min between individual runs. Flow rate, injection volume, and column and temperature were as follows: 0.4 ml/min, 5 μl, and 40°C, respectively.

UV‐VIS spectra of phenolic compounds were recorded from 190 to 400 nm, and the samples were detected at 280 for phenolic acids and 335 nm for flavonoids. Quantitative evaluation of phenolic compounds was performed in comparison with its standard when it was available by means of a four‐point regression curve (*r* ^2^ = .989) using authentic external standards.

### 2.8 Antioxidant activity

#### 2.8.1 DPPH radical scavenging activity

The free radical scavenging capacity was measured using the DPPH method described in a previous study (Ben Hmed et al., 2020; Khedher et al., 2020). Aliquots (1 ml) of various dilutions of the methanol‐aqueous extracts were mixed with 4 ml of methanolic DPPH solution (0.2 mM). The mixtures were incubated for 30 min at 25°C, and then, the absorbance at 517 nm was measured using a UV‐Visible spectrometer (BECKMAN DU 800). The absorbance in the presence of methanol‐aqueous extract was recorded as *A*_sample_ while the absorbance of the control reaction was recorded as *A*_blank_. The free radical scavenging activity of each solution was then calculated as inhibition percentage as follows Equation (1):

(1) $$\%\;\;\text{Inhibition = [}\frac{A_{\text{blank}}‐A_{\text{sample}}}{A_{\text{blank}}}]\times 100$$

Antiradical activity was expressed as IC_50_ (mg/ml), defined as the concentration of the extract required to cause a 50% decrease in initial DPPH concentration. All measurements were performed in triplicate.

#### 2.8.2 ABTS radical cation scavenging activity

The ABTS radical scavenging activity of the *pinus halepensis* seed extracts was performed using the method by Rigane et al. (2011). Briefly, ABTS was dissolved in water to get a 7 mMconcentration. ABTS radical (ABTS.^+^) was produced by reacting this stock solution with a 2.45 mMK_2_S_2_O_8_ solution and allowing the mixture to stand in the dark at room temperature for 12–16 hr before use. The ABTS.^+^ solution was diluted with methanol to an absorbance of 0.70 ± 0.02 at 730 nm. After the addition of 100 µl of sample, methanol as a blank, or Trolox standard to 2.9 ml of diluted ABTS.^+^ solution, absorbance readings were taken after 6 min. The antioxidant capacities of the *Pinus halepensis* seeds extracts were evaluated using a standard curve obtained by measuring the absorbance of Trolox solutions (0.02–0.08 mM).

### 2.9 Statistical analysis

Results were expressed as mean ± *SD* of 3 measurements for the analytical determination. Statistical differences were calculated using a one‐way analysis of variance (ANOVA), followed by Student's *t* test. Differences were considered significant at *p* < .05.

## 3 RESULTS AND DISCUSSION

### 3.1 Determination of minerals

The main objective of this section was to study the mineral composition of *Pinus halepensis* cones dried by two different methods. As shown in Table 1, calcium, potassium, sodium, and manganese were the predominant mineral elements in the two studied samples with a significant differences between the cones’ dried methods (*p* < .05). These elements are essential for its biological functions as well as they are very important for health especially for K and Na which were necessary for the acid–base balances especially in the intra‐cellular fluid and functions as well as in conduction of nerve impulses, muscle contraction, and Na^+^/K^+^‐ATPase (Kadri et al., 2015; Yahyaoui et al., 2019).

**TABLE 1:** Some physical and chemical characteristics of *Pinus halepensis* Mill. seeds

	Convection‐drying method	Sun‐drying method
Oil content	17.06 ± 0.1^a^	22 ± 0.2^b^
Minerals (mg/100g)		
Calcium	54.04 ± 0.05^a^	67.89 ± 0.07^b^
Magnesium	2.84 ± 0.02^a^	3.01 ± 0.03^b^
Potassium	176.23 ± 0.01^a^	197.88 ± 0.01^b^
Sodium	100.865 ± 0.07^a^	223.88 ± 0.07^b^
Iron	14.88 ± 0.05^a^	16.37 ± 0.06^b^
Zinc	7.69 ± 0.04^a^	7.93 ± 0.04^a^
Manganese	195.04 ± 0.07^a^	223.69 ± 0.02^b^
Total phenolic content (mg GAE/g DW)	12.69 ± 0.07^a^	14.63 ± 0.05^b^
Total flavonoid content (mg QE/g DW)	2.70 ± 0.01^a^	3.3 ± 0.02 ^b^
Condensed tannins content (mg CE/g DW)	0.35 ± 0.05^a^	0.36 ± 0.05^b^
DPPH assay (mg/ml)	0.1 ± 0.13^a^	0.08 ± 0.00 ^b^
ABTS assay (mg/ml)	0.08 ± 0.00^a^	0.05 ± 0.00 ^b^

Note

Results are expressed as mean ± *SD* of three determinations. Means with different letters in the same line were significantly different at *p* < .05.

Abbreviation: DW: dry weight.

### 3.2 Oil content

Oil extracted from *P. halepensis* cones dried with two different methods has been evaluated (Table 1). We found that dried *P. halepensis* cones with convection method allow us to recovery low seeds’ oil content (17.06 ± 0.1%) in comparison with those obtained using sun‐dried method (22 ± 0.2%). The late results were in accordance with those reported by Chaabenet al., (2015), who have claimed that *P. halepensis* in Tunisia contains about 21.6 ± 0.1 of oil and to those presented, by Rigane and co‐workers in 2019, when they studied the chemical composition of *P. halepensis* Mill. oil obtained from two different locations: Kasserine (17.6%) and Foussena (22%). Other previous studies have reported high oil content in same *specie* with 43.3% (Cheikh‐Rouhou et al., 2006) and in general, the rates of Tunisian pine seeds’ oil varied between 34.63% and 48.12%. On the other hand, Algerian *Pinus* seeds were characterized by oil content ranged from 19.78% to 36.73% for *P. pinea* L. and *P. halepensis* Mill., respectively (Kadri et al., 2015). Therefore, the difference between pine seeds’ oil content depends especially to the pines *specie,* geographic variation, as well as environmental conditions (Kadri et al., 2015; Nasri et al., 2005; Rigane et al., 2019).

### 3.3 Fatty acid composition

**FIGURE 2:**
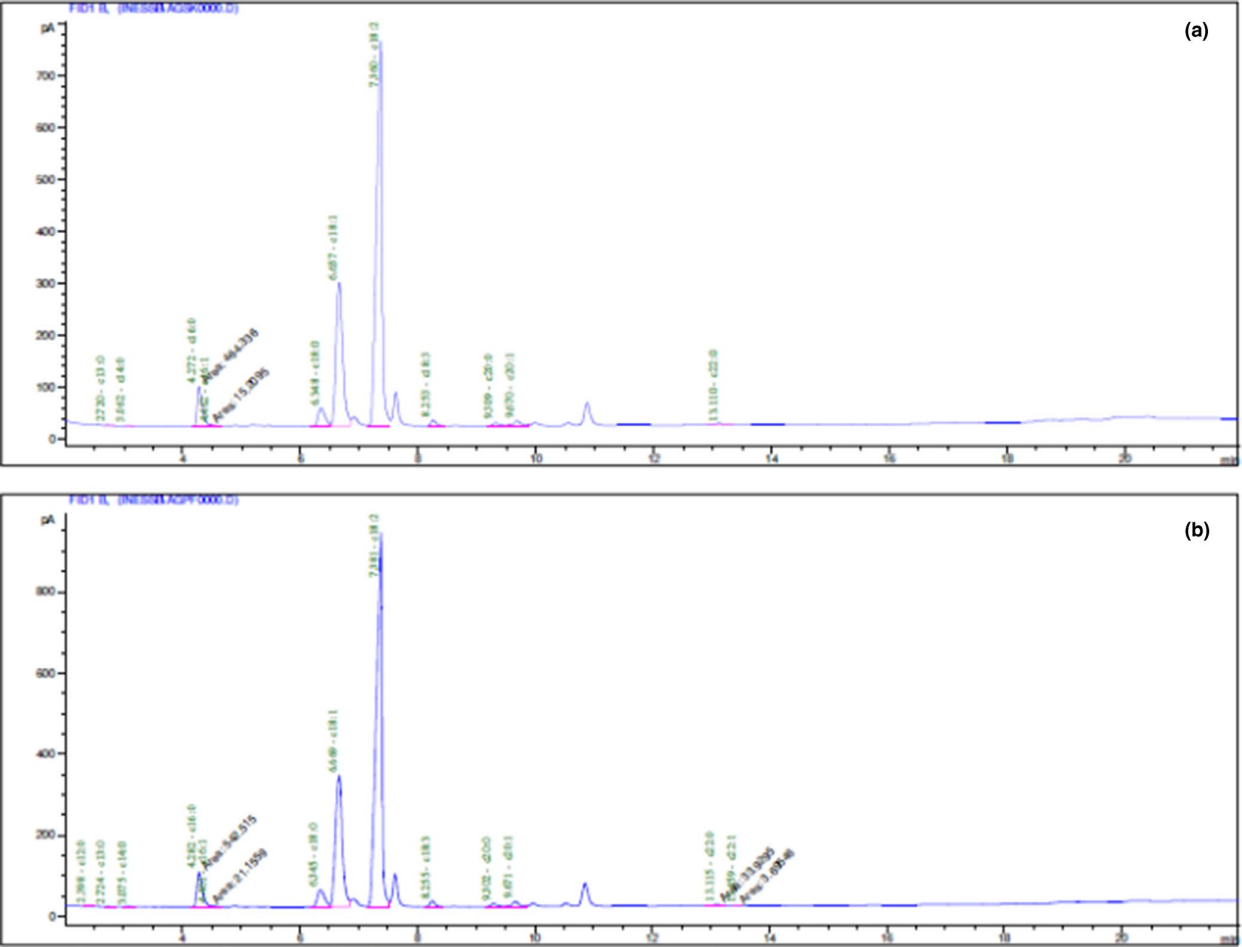
GC‐FID chromatograms of fatty acid composition of *Pinus halepensis* Mill. oil cones dried by convention (a) and sun (b) methods

The chemical composition of the fatty acids of cones was investigated using GC‐FID apparatus. The obtained chromatograms of *P. halepensis* Mill. oil seeds dried by convection and sun methods showed that the studied oils were a mixture of numerous fatty acids; some of them were present in trace amounts (Figure 2).

The results showed that the fatty acids present in the oils are mainly UFAs for 89.74% in oil seeds obtained after convection‐drying method and 90.10% for oil seeds obtained after sun‐drying method (Table 2). The last oil was characterized by high‐level linoleic acid (62.50%); which may be decreased dramatically its oxidative stability, followed by oleic acid (26.30%) while linolenic acid level did not exceed 1%. For oils obtained from the two studied dried methods, the saturated fatty acid level was near to 10%; this could be explained by the presence of high quantities of palmitic acid (~6%). From these results, we showed that drying cones’ methods affect significantly on the fatty acid percentages (*p* < .05).

**TABLE 2:** Fatty acid composition of *Pinus halepensis* Mill. seeds

	Convection‐drying method	Sun‐drying method
Lauric acid (C12:0)	trace	trace
Tridecylic acid (C13:0)	0.02 ± 0.00^a^	0.02 ± 0.00^a^
Myristic acid (C14:0)	0.05 ± 0.00^a^	0.05 ± 0.00^a^
Palmitic acid (C16:0)	5.73 ± 0.05^a^	5.37 ± 0.01^b^
Hypogeic (C16:1n‐9) + palmitoleic (C16:1n‐7) acids	0.18 ± 0.03^a^	0.20 ± 0.06^b^
Stearic acid (C18:0)	3.52 ± 0.02^a^	3.59 ± 0.00^b^
Z‐vaccenic (C18:1n‐7) + Oleic (C18:1n‐9) acids	27.00 ± 0.01^a^	26.30 ± 0.02^b^
Linoleic acid (C18:2)	60.91 ± 0.07^a^	62.50 ± 0.09^b^
Linolenic acid (C18:3)	0.75 ± 0.00^a^	0.76 ± 0.00^b^
Arachidic acid (C20:0)	0.55 ± 0.01^a^	0.55 ± 0.02^a^
Gadoleic acid (C20: 1n‐9)	0.90 ± 0.06a	0.33 ± 0.05^b^
Behenic acid (C22:0)	0.34 ± 0.02^a^	0.21 ± 0.06^b^
Erucic acid (C22:1)	trace	0.01 ± 0.00
C18:1 / C18:2	0.44 ± 0.04^a^	0.42 ± 0.09^b^
∑ SFAs	10.21 ± 0.10^a^	9.79 ± 0.09^b^
∑ MUFAs	28.08 ± 0.10^a^	26.84 ± 0.05^b^
∑ PUFAs	61.66 ± 0.07^a^	63.26 ± 0.09^b^
∑ UFAs	89.74 ± 0.17^a^	90.10 ± 0.14^b^
∑ MUFAs / ∑ PUFAs	0.45 ± 0.54^a^	0.42 ± 0.08^b^

Note

Results were expressed as mean ± *SD* of 3 determinations. Means with different letters in the same line were significantly different at *p* < .05.

Abbreviations: MUFAs, monounsaturated fatty acids; PUFAs, polyunsaturated fatty acids; SFAs, saturated fatty acids; UFAs, unsaturated fatty acids.

Furthermore, our results were similar to those reported by Kadri et al. (2015) and Nergiz and Domnez (2004) who mentioned that oleic and linoleic acids at relatively high levels in many *Pinus* varieties’ oils, while Rigane et al. (2019) did not identified and quantified linoleic acid when they studied the fatty acid composition of *P. halepensis* Mill. oil growing in Kasserine center and Foussana countryside. These two fatty acids are essential for healthy growth of human skin and in the construction of the nerve cells (Kadri et al., 2015). On the other hand, our results were slightly different from those found in Tunisian and Turkish *P. halepensis* Mill. oils. For example, Tunisian *P. halepensis* Mill. oil contained 48.8% of linoleic acid and 27.3% of oleic acid (Cheikh‐Rouhou et al., 2006) while the linoleic acid was found as the main UFA in the *P. pinea* L. seeds oils growing in Turkey (47.6%) and Tunisia (47.28%) (Nasri et al., 2005; Nergiz & Domnez, 2004).

Therefore, and taking into account the obtained results, we can conclude that drying *P. halepensis* Mill. cones by convention methods was the appropriate method to get healthy oil. In addition, the results of this study could be interesting for *P. halepensis* oil packagers and marketers to estimate the caducity date of *P. halepensis* quality oil.

### 3.4 Total phenolic, flavonoids, and condensed tannin contents

As shown in Table 1, the total phenol content was 14.63 ± 0.05 and 12.69 ± 0.07 mg GAE/g DW of *P. halepensis* Mill. seeds dried in Sun and convection methods, respectively. The same tendency was observed in the content of flavonoids and condensed tannins. Therefore, the total phenolic, flavonoids as well as condensed tannin contents in the methanolic‐aqueous seeds extract of *P. halepensis* Mill. varied significantly between the two drying methods (*p* < .05). Our results were not similar to those reported by Kadri et al., (2015), who found low total phenolic contents in four Algerian pine seeds extracted with methanol: *P. halepensis* Mill., *P. pinea* L., *P. pinaster,* and *P. canariensis*. The Algerian research team mentioned that phenolic content varied between 3.71 and 9.37 mg GAE/g DW. On the other hand, for the study made by Ustun et al. (2012), Aleppo pine seeds of Turkish origin gave a value equal to 72.77 mg GAE/g DW for the ethanolic extract against 102.56 mg GAE/g DW for the extract extracted by ethyl acetate. In addition, Siberian pine “*P. sibirica*” and Korean pine “*P. koraiensis*” (266 and 264 mg/g, respectively) were characterized by its high total phenolic content in comparison with many Aleppo pine seeds *species* growing in the North of African (Lantto et al., 2009; Su et al., 2009).

### 3.5 LC‐ESI‐MS analysis

**FIGURE 3:**
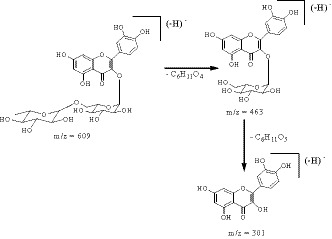
Proposed fragmentation from the pseudomolecular anion of compound **4**

**FIGURE 4:**
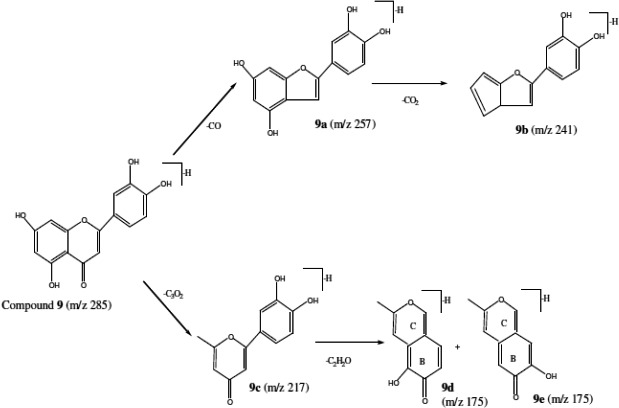
Proposed fragmentation from the pseudomolecular anion flavone luteolin

LC‐ESI‐MS analysis was performed in order to increase the nutritional value of *P. halepensis* Mill. The identification of the phenolic compounds was carried out by mass spectra, comparison with reference compounds and with literature data (Rigane et al., 2011; Rigane et al., 2013; Boukhris et al., 2013; Ben Salah et al., 2019; Ben Hmed et al., 2020). Table 3 illustrates all the identified peaks with retention times, the pseudomolecular ions, as well as the concentration of each identified phenolic compound. Eleven phenolic compounds were tentatively identified in *P. halepensis* Mill. methanolic‐aqueous extract including two phenolic acids: protocatechuic acid and *trans*‐ferulic acid, and nine flavonoids: luteolin‐7‐*O*‐glucoside, kaempferol, catechin (+), cirsiliol, cirsilineol, naringin, luteolin, apigenin, and rutin. For example, compound **4** (*t*_R_ 24.617 min) was identified as quercetin aglycon that was assigned according to the presence of a main peak at m/z 609 as well as a strong peak at m/z 301 in its ESI‐mass spectrum at negative mode. The compound **4** MS^2^ mass spectrums showed fragments at m/z 463 [M‐H‐rhamnosyl]^−^ and 301[M‐H‐rhamnosyl‐glucosyl]^−^ (Figure 3). These results revealed that compound **4** was a rutin (Ben Salah et al., 2019). In addition, compound **6** had a deprotonated molecule [M‐H]^‐^ at m/z 447, and a strong fragment at m/z 285 suggested that this compound was the luteolin‐7‐*O*‐glucoside. While mass spectrum of compound **8** showed a high‐intensity ion fragment at m/z 269, the ESI‐MS^2^ spectrum of that ion showed m/z at 225 [M‐H‐44]^‐^ and 201 [M‐H‐68]^‐^. The m/z 225 can be explained by the loss of CO_2_ molecule from A ring, while the m/z 201 was obtained by the elimination of C_3_O_2_ from β‐dihydroxy in A ring. The obtained mass spectra suggested that compound **8** was apigenin. Furthermore, the MS analysis of compound **9**, luteolin, showed a predominant [M‐H]^‐^ signal at m/z 285 whose MS^2^ fragmentation spectrum indicates m/z at 257 [M‐H‐CO]^‐^, 241 [M‐H‐CO_2_]^‐^, 217 [M‐H‐C_3_O_2_]^‐^, and 175 [M‐H‐C_3_O_2_‐C_2_H_2_O]^‐^. The loss of 28 and 44 Da could be justified by the elimination of CO (9a) and CO_2_ (9b) from C and A ring, respectively (Figure 4). Moreover, the fragment m/z at 217 obtained by C_3_O_2_ loss implies unambiguously the β‐dihydroxy configuration displayed by the A ring (9c). The resulting 9c fragment proposed (Figure 4) includes a methyl group and undergoes further C_2_H_2_O loss leading to two possible fragments (9d and 9e). These results were in accordance with the proposed scheme of fragmentation in some flavones aglycon by Fabre et al. (2001).

**TABLE 3:** Phenolic compounds detected in *Pinus halepensis* Mill. seeds extracts

No	Compounds	Retention time (min)	Molecular mass	[M‐H]^−^ m/z	Convection‐drying method^a^	Sun‐drying method^a^
**1**	Protocatechuic acid	7.350	154	153	0.264 ± 0.02^a^	0.219 ± 0.01^b^
**2**	Catechin (+)	12.094	290	289	0.569 ± 0.01^a^	0.888 ± 0.03^b^
**3**	*trans*‐Ferulic acid	24.050	194	193	0.091 ± 0.00^a^	0.135 ± 0.00^b^
**4**	Rutin (quercetin−3‐*O*‐rutinoside)	24.617	610	609	0.038 ± 0.00^a^	0.038 ± 0.00^a^
**5**	Naringenin	26.861	272	271	ND	0.014 ± 0.00
**6**	Luteolin−7‐*O*‐glucoside	27.650	448	447	0.017 ± 0.00^a^	0.148 ± 0.00^b^
**7**	Kaempferol	32.916	286	285	0.022 ± 0.00^a^	0.028 ± 0.00^a^
**8**	Apigenin	35.400	270	269	ND	0.001 ± 0.00
**9**	Luteolin	35.617	286	285	0.589 ± 0.00 ^a^	1.760 ± 0.00^b^
**10**	Cirsiliol	36.390	330	329	1.916 ± 0.03 ^a^	0.761 ± 0.00^b^
**11**	Cirsilineol	39.750	344	343	ND	0.043 ± 0.00

Note

Abbreviations: DW, dry weight; ND, not detected.

Results are expressed as mean ± *SD* of three determinations. Means with different letters in the same line were significantly different at *p* < .05.

^a^ Concentration expressed as mg/100 g of DW.

**FIGURE 5:**
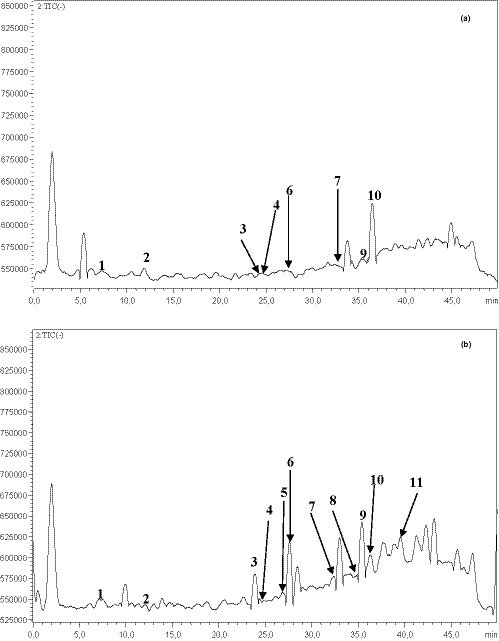
HPLC chromatograms of phenolic composition of *Pinus halepensis* Mill. oil cones dried by convention (a) and Sun (b) methods. **1**: Protocatechuic acid, **2**: catechin (+), **3**: *trans*‐Ferulic acid, **4**: rutin (quercetin‐3‐*O*‐rutinoside), **5**: naringenin, **6**: luteolin‐7‐*O*‐glucoside, **7**: kaempferol, **8**: apigenin, **9**: luteolin, **10**: cirsiliol, **11**: cirsilineol

The amount of individual phenolic compounds, as summarized in Table 3, showed an important difference between the Sun and the convection‐drying methods. In this study, we reported for the first time the phenolic phytochemical profile analysis of *P. halepensis* Mill. methanolic‐aqueous extract obtained from two different drying methods (Figure 5). Our results showed that there were significant differences between Sun and convection‐drying methods (*p* < .05). Table 3 revealed the presence of *trans*‐ferulic and protocatechuic acids in the two studied samples (0.091–0.135 and 0.219–0.264 mg/100 g of DW, respectively). From Table 3, we could conclude that cirsiliol was the major flavonoids compounds quantified in the *Pinus halepensis* Mill. seeds (0.761–1.916 mg/100 g of DW), followed by luteolin (0.589–1.760 mg/100 g of DW), catechin (+) (0.569–0.888 mg/100 g of DW), and luteolin‐7‐*O*‐glucoside (0.017–0.148 mg/100g of DW). On the other hand, cirsilineol, naringenin, and apigenin were present only in extracts obtained from *Pinus halepensis* Mill. seeds dried in Sun (≤0.043 mg/100 g of DW). From these results, our research team concluded that this study could provide useful information for industry to produce the potentially bioactive compound extracted from *P. halepensis* Mill. seeds using convection or sun‐drying method on the phenolic composition.

### 3.6 Impact of drying methods on antioxidant activities

To compare the antioxidant activities of the two *P. halepensis* Mill. seed extracts, DPPH and ABTS radical scavenging activities were tested, being the results presented in Table 1. Measurements of antioxidant activity of the two methanolic‐aqueous extracts showed a significant variation between the two dried seeds methods (*p* < .05). Regarding the values of the antioxidant capacity of the studied samples, the highest antiradical activities were enregistred in *P. halepensis* Mill. cones dried using sun method (0.08 and 0.05 mg/ml, for DPPH and ABTS, respectively). These results could be explained as reported previously by Chouaibi et al. (2019) who claimed that the complex component and synergistic effects of antioxidant compounds could be attributed to this high antioxidant activity against 2,2‐diphenyl‐1‐picrylhydrazyl and 2, 2´‐azinobis (3‐ethylbenzothiazoline)‐6‐sulfonic acid radicals.

## 4 CONCLUSION

With the aim of studying the effect of *P. halepensis* Mill. cones drying methods including convection and sun methods, the results showed that fatty acid content, total phenolic, flavonoids, and condensed tannin contents as well as mineral composition were significantly (*p* < .05) influenced by the methods used in the drying of *P. halepensis* Mill. cones. As a general statement, it can be concluded that seed cones dried using sun method have the best mineral and phenolic composition while the best fatty acid composition was obtained from seeds oil cones dried by convection method. The antioxidant activities of seed extracts obtained from dried cones by sun method could be accredited to the high content of phenolic and flavonoids components identified in *P. halepensis* Mill. seeds by HPLC‐MS.

## CONFLICT OF INTEREST

The authors hereby declare that there are no conflicts of interest.

## AUTHOR CONTRIBUTIONS

Amani Mahdhi did practical experiences, wrote, followed, and checked the obtained results. Meriem El Aloui did some practical experiences, coordinated all the analysis, and calculated the results, statistics, etc. Hanene Ghazghazi, Ridha Ben Salem, and Ghayth Rigane supervised the scientific paper.

## ETHICAL APPROVAL

This article does not contain any studies with human participants or animals performed by any of the authors.

### INFORMED CONSENT

Informed consent not applicable.

## Data Availability

The data that support the findings of this study are available from the corresponding author upon reasonable request.
